# Characterization of Post-Viral Infection Behaviors Among Patients With Long COVID: Prospective, Observational, Longitudinal Cohort Analyses of Fitbit Data and Patient-Reported Outcomes

**DOI:** 10.2196/77644

**Published:** 2025-12-31

**Authors:** Tianmai M Zhang, Sydney P Sharp, John D Scott, Douglas Taren, Jane C Samaniego, Elizabeth R Unger, Jeanne Bertolli, Jin-Mann S Lin, Christian B Ramers, Job G Godino

**Affiliations:** 1 Laura Rodriguez Research Institute Family Health Centers of San Diego San Diego, CA United States; 2 Department of Biomedical Informatics and Medical Education University of Washington Seattle, WA United States; 3 Department of Medicine University of Washington Seattle, WA United States; 4 Department of Pediatrics Nutrition Section University of Colorado Aurora, CO United States; 5 National Center for Emerging and Zoonotic Infectious Diseases Centers for Disease Control and Prevention Atlanta, GA United States; 6 School of Medicine University of California, San Diego La Jolla, CA United States; 7 School of Public Health San Diego State University San Diego, CA United States; 8 Global Hepatitis Program Clinton Health Access Initiative Boston, MA United States; 9 Herbert Wertheim School of Public Health and Human Longevity Science University of California, San Diego La Jolla, CA United States; 10 Exercise and Physical Activity Resource Center University of California, San Diego La Jolla, CA United States

**Keywords:** long COVID, objective physical activity, phenotype, wearable device, Fitbit, post-viral infection behaviors, mobile phone

## Abstract

**Background:**

Long COVID encompasses a range of health problems that can be highly debilitating. While some research has relied on self-reported measures of symptoms and functioning, few studies have characterized symptoms in relation to behaviors and physiology measured objectively through wearable devices.

**Objective:**

The primary aim of this study was to identify longitudinal patterns in physical activity, physiology, and patient-reported outcomes (PROs) among patients with long COVID at a Federally Qualified Health Center in the United States. The secondary aim was to identify meaningful subgroups or phenotypes within this cohort and examine how PROs and symptoms overlay with physical activity characteristics.

**Methods:**

This was a prospective, observational, longitudinal cohort study recruiting a subset of low-income patients enrolled in the Long COVID and Fatiguing Illness Recovery Program. From March 2022 to May 2023, a total of 172 patients with long COVID or myalgic encephalomyelitis/chronic fatigue syndrome were given Fitbit Charge 5 (Fitbit Inc) devices and instructed to wear them continuously for up to a year. Patients completed PRO questionnaires (PROMIS-29 [Patient-Reported Outcomes Measurement Information System-29] and symptom questionnaires, etc) at baseline, 3, and 6 months. Inclusion in longitudinal analysis required at least 20 hours of valid wear data per day for a minimum of 7 days. The World Health Organization guideline on moderate to vigorous physical activity (MVPA) was used to differentiate MPVA-active and MVPA-inactive patients. Linear mixed effects regression was performed to assess longitudinal associations between physical activity levels and PROs.

**Results:**

Among 172 patients, 80.2% (n=138) were female, 75.6% (n=130) were White, 45.3% (n=78) were unemployed, and 94.8% (n=163) had estimated annual income below US $50,000. Of these patients, 82 (47.7%) met valid wear criteria, providing 50.5 days of Fitbit data on average. At baseline, MVPA-inactive patients (n=41) reported statistically more severe problems regarding physical function, fatigue, and dyspnea than MVPA-active patients (n=41) on both continuous and categorical scales, with *P*<.05 from both Student *t* tests (2-tailed) and chi-squared tests. Longitudinal analysis found that MVPA-active patients had statistically less improvements in the ability to participate in social roles (estimated group difference=–4.21 T-score points over 3 months, 95% CI –6.64 to –1.78, *P*<.001) and a higher intensity of sleep symptoms (estimated group difference=2.06 severity score points over 3 months, 95% CI 0.40 to 3.71, *P*=.02) over time.

**Conclusions:**

This study showed that patients with long COVID could remain MVPA-active despite experiencing symptoms. These findings provide insights into the relationship between PROs, physical activity, and long COVID, which suggests the importance of considering individual activity profiles when tailoring treatment plans, especially in a low-income population. The findings of this study should be interpreted as hypothesis-generating, considering its exploratory nature and limitations, including high attrition rates and missing data.

## Introduction

COVID-19 has resulted in over 100 million infections and over 1 million deaths in the United States alone [1,2]. Patients who are infected with SARS-CoV-2 can experience a range of highly diverse clinical presentations, severities, and outcomes ranging from no symptoms to critical illness [3,4]. Some patients report lasting, new, or recurring symptoms and conditions more than 4 weeks after infection, commonly known as long COVID [4]. The scientific understanding of diagnoses, epidemiology, and phenotypes of long COVID is continually evolving; however, there is still much to be discovered and investigated [5]. Notably, there was not a consistent definition for long COVID until the National Academies of Sciences, Engineering, and Medicine recently defined it as an “infection-associated chronic condition (IACC) that occurs after SARS-CoV-2 infection and is present for at least 3 months as a continuous, relapsing and remitting, or progressive disease state that affects one or more organ systems” [6].

Long COVID encompasses a range of health problems and symptoms that may be highly debilitating and may arise secondary to detectable organ system damage, autoimmunity, and other mechanisms [6,7]. The recent Household Pulse survey estimated that among people who currently have long COVID, around 25% have significant activity-limiting symptoms [8]. In many patients, the etiopathogenesis is unclear [9]. As of September 2024, it was estimated that 18% of United States adults had experienced long COVID [8]. Typical symptoms associated with long COVID, such as cognitive impairment, fatigue, and post-exertional malaise (PEM), closely resemble those in patients diagnosed with myalgic encephalomyelitis/chronic fatigue syndrome (ME/CFS) and other IACCs [10,11].

Increased risk of long COVID is associated with being female, experiencing socioeconomic deprivation, and being a member of an ethnic minority group [12,13]. Furthermore, individuals with lower incomes face greater stress, poorer access to health care, and environmental exposures compared to those with higher incomes [14]. These disparities highlight the need to study long COVID in medically underserved populations rather than in specialty clinics [15]. As California alone has about 5.9 million low-income residents, it is important to understand long COVID-related symptom experiences, physiological characteristics, and behavioral patterns in this population, which may support future improvements in the delivery of care.

Symptoms associated with long COVID are highly variable and are often unpredictable. An individual may experience day-to-day variation in symptoms and their severity [16,17]. Similarly, the way an individual responds to an event or stimulus is highly individualized and can often result in different behavioral outputs, depending on the day [18]. As the clinical presentation, severity, and outcomes of long COVID vary from patient to patient, it may prove beneficial to monitor the disease in a more personalized way, for example, through a wearable device.

Wearable devices that continuously track behavioral and physiological metrics such as steps, heart rate (HR), heart rate variability (HRV), temperature, physical activity, and sleep have become more accurate and accessible. It is estimated that one in three Americans uses a wearable device to help track their fitness and health [19]. Several studies and reviews have found that several wearable devices have demonstrated utility in infectious-disease surveillance, including early detection of COVID-19 and the monitoring of physiological signals pre and post infection [20-25].

As the wearable research landscape shifted from early detection to management of long COVID, several studies and reviews assessed a variety of wearables and approaches to identify persistent physiological clusters, support pacing or self-management, and track recovery trajectories [26,27]. However, the literature has a limited representation of low-income populations. Although many studies incorporated patient-reported outcomes (PROs), few used a longitudinal design to examine symptom trajectories alongside objective activity measures, and none did so in a Federally Qualified Health Center (FQHC) cohort.

To address this gap, we collected high-resolution longitudinal data from a subset of patients in the Long COVID and Fatiguing Illness Recovery Program (LC&FIRP) through Family Health Centers of San Diego (FHCSD) using the Fitbit Charge 5 (Fitbit Inc). This study sought to answer the following research questions: (1) What longitudinal characteristics do patients with long COVID have in terms of Fitbit-measured physical activity, physiology, and PROs? (2) Are there any meaningful subgroups or phenotypes within this patient cohort? (3) Are there longitudinal associations between the identified phenotypes and the changes in PROs that could inform future studies and clinical management of long COVID?

## Methods

### Study Design and Setting

This was a prospective, observational, longitudinal cohort study involving a subset of patients enrolled in LC&FIRP. LC&FIRP was a three-year effectiveness-implementation hybrid study at an FQHC, FHCSD. FHCSD is one of the ten largest FQHC health systems in the nation, with the vast majority of its patients being low-income and members of a minority population [28]. The program used a technology-enabled multidisciplinary team-based care model focused on case-consultation and peer-to-peer discussion of emerging best practices (ie, teleECHO [Extension for Community Healthcare Outcomes]) to assist management of complex cases associated with long COVID, ME/CFS, and other IACCs [28]. This study was reported in accordance with the STROBE (Strengthening the Reporting of Observational Studies in Epidemiology) guidelines [29]. The STROBE checklist of this study is available in Multimedia Appendix 1.

### Aims

The primary aim of this study was to identify longitudinal patterns in physical activity, physiology, and PROs of patients with long COVID. The secondary aim was to identify meaningful subgroups or phenotypes within this study’s cohort and examine the overlay of PROs and symptoms in relation to the activity characteristics of patients with long COVID.

### Patients

Between March 2022 and May 2023, Fitbit devices for remote monitoring and symptom management were given to a subset (n=172) of the total enrolled LC&FIRP patient cohort (n=590). The sample size was based on 20% of LC&FIRP’s initial recruitment target of 856. Using convenience sampling, participants were recruited when they agreed to complete their physical therapy visit or through a provider or physical therapist referral. Inclusion criteria included being aged 18 years or older, documented persistent symptoms and a decline in health-related quality of life consistent with long COVID based on patient report and clinical determination, ME/CFS or other IACCs, and having a smartphone. No additional measures were implemented to address potential sources of bias beyond standard study methods.

Eligible patients who completed their baseline survey and were willing to participate in the Fitbit substudy received Fitbit Charge 5 devices within 3 months. Participants also received a booklet explaining how to interpret Fitbit data to manage their symptoms using pacing. An activity diary was also included in the back of the booklet to encourage patients to record their activities and symptoms for a week straight. Patients were offered an incentive to submit the activity diary; however, only 4 patients submitted diaries, and therefore, these data are not included in the current analysis.

Patients completed PROs including the PROMIS-29 (Patient-Reported Outcomes Measurement Information System-29) version 2.1, the PROMIS (Patient-Reported Outcomes Measurement Information System) Short Form version 1.0 - Dyspnea Severity 10a and Applied Cognitive General Concerns 8a, the PHQ-2 (Patient Health Questionnaire-2), the GAD-7 (Generalized Anxiety Disorder-7), and the Centers for Disease Control and Prevention (CDC) ME/CFS Symptom Screener-Short Form version 1.2 at baseline, 3 months and 6 months [30-35]. They were completed over the phone and in English or Spanish, depending on the patient’s preferred language. During follow-up, patients were only asked about symptoms they reported at baseline; however, patients had the opportunity to report any newly experienced symptoms during each follow-up. Data regarding HR, sleep, and physical activity were tracked by Fitbit devices in real time for up to a year and automatically uploaded to Fitabase (Small Steps Labs LLC), a Fitbit application programming interface partner that provided a platform for FHCSD to view clean data. After the 6-month survey date of the last patient in December 2023, Fitbit data was downloaded from Fitabase and preprocessed for analysis.

To minimize loss to follow-up, the research team contacted patients at least three times over 4 weeks to complete their surveys. The team also reviewed Fitabase weekly to confirm Fitbit data uploads. If a patient had not uploaded data for a week or more, researchers made three calls or text attempts over the next 4 weeks to remind them to wear the Fitbit and open the app to enable data upload.

### Ethical Considerations

LC&FIRP was reviewed and approved by the Institutional Review Board of San Diego State University (HS-2021-0241). All methods were carried out in accordance with relevant guidelines and regulations, and present no more than minimal risk to patients. All patients included in this study completed FHCSD’s Broad Consent, which includes a specific authorization for the use of deidentified health information for research purposes, including primary data collection and analyses. Any information obtained about patients during this study was deidentified and treated as strictly confidential to the full extent permitted by applicable law and in accordance with Health Insurance Portability and Accountability Act regulations. All data is deidentified and reported in aggregate. More information regarding data privacy and management can be found in the study protocol [28]. Patients kept the Fitbit Charge 5 devices they received after the current study as compensation for their participation in the Fitbit substudy, and a US $10 gift card was provided as an incentive for completing and returning the activity diary. No additional compensation was provided.

### Data Preprocessing

Preprocessing of minute-level Fitbit data followed the recommendations of Wing et al [36]. Specifically, minutes with valid HR values were automatically considered valid wear. For minutes with missing HR data but with corresponding step counts, metabolic equivalent of task (MET), or intensity values above default (ie, step count above 0, MET above 1.0, or intensity above 1), the mean HR value from adjacent minutes was used to fill in gaps [36]. Next, minutes with HR values below 45 or above 205 (0.23% and <0.001% of total observed minutes, respectively) were excluded due to their improbable occurrence. Additionally, periods where the HR value was unchanged for 11 or more consecutive minutes (1.39% of all observed minutes) were also excluded because they are unlikely to be physiological [36]. Subsequently, the minute-level time series data were aggregated into daily summaries to determine valid daily wear time and daily features of each participant. Summary measures for characterizing daily behaviors of patients were calculated from available Fitbit measurements. To retain longitudinal patterns in the Fitbit data, we did not conduct data imputation in consideration of the large proportion of missing data.

PRO scores were calculated according to their respective protocols. Severity scores of the 8 symptoms assessed by the CDC ME/CFS Symptom Screener were calculated according to the scoring algorithm of the CDC Symptom Inventory [34,35], which takes into account both the frequency and intensity of a symptom. Analyses of PROMIS scores were based on both raw scores and T-scores (ie, standard scores with a mean of 50 and SD of 10 in the US general population) calculated using the HealthMeasures Scoring Service online [37], while all other processes were performed using Python (version 3.8.15; Python Software Foundation). The Hawkins test suggested that the missingness pattern of the PRO data is consistent with a missing completely at random mechanism, with *P*>.10 for all PRO measures.

### Patient Categorization

In this study, a valid wear day is defined as having at least 1200 minutes (ie, 20 hours) of valid Fitbit data. Patients are considered to have valid wear during this study if they have had at least 7 valid wear days among all available data.

Inspired by the findings from exploratory time series analysis that patients could be classified into 2 clusters by their moderate to vigorous physical activity (MVPA), we further categorized valid wear patients as MVPA-active or MVPA-inactive based on the World Health Organization (WHO) guideline of 150 MVPA minutes per week [38]. MVPA refers to the physical activity that is performed at >3 METs (ie, >3 times the intensity of rest) on an absolute scale [38]. Due to the discontinuous nature of valid wear days, we calculated average daily MVPA minutes of each patient between baseline and 6 months and categorized patients as MVPA-active or MVPA-inactive by whether their average daily MVPA reached 21.4 minutes.

### Statistical Analysis

Statistical differences between subgroups of patients (eg, stratified by Fitbit wear time or MVPA) at different time points were examined using the chi-squared test, Student *t* test, or Wilcoxon test, according to data type and normality. Two-way ANOVA was used to examine group differences while adjusting for another factor of interest (eg, employment status).

For exploratory analysis of potential patient clusters, growth mixture modeling (GMM) [39] was applied on day-level time series of Fitbit measures.

Linear mixed effects (LME) [40] regression was used to model longitudinal associations between MVPA levels and PROs, using Fitbit data between baseline and 6 months. LME models of PROs included time (baseline, 3-month, and 6-month), MVPA levels, the interaction between time and MVPA levels, and baseline PRO scores, plus an individual intercept for each patient to account for random effects. The interaction term would reveal the effect of MVPA level over time. Models with different sets of independent variables or different random effect settings were evaluated with the Akaike Information Criterion to find the best model. Model validity was diagnosed using the Shapiro-Wilk test of residual normality and White Lagrange multiplier test of residual variance homogeneity. Sensitivity analysis was subsequently performed to assess the validity of statistically significant findings, using slightly different thresholds for MVPA classification, different lengths of Fitbit data, categorical time labels, and different patient inclusion strategies.

GMM was performed using the *lcmm* package [41] (version 2.1.0) in R (version 4.3.3; R Foundation); LME was performed using *lmerTest* [42] (version 3.1-3) in R (version 4.3.3); other analyses were performed using SciPy (version 1.10.1) and statsmodels (version 0.14.0) in Python (version 3.8.15). A *P* value of less than .05 is considered indicative of a statistically significant difference or association. Due to the exploratory, hypothesis-generating nature of our analyses, we did not make adjustments for multiple testing.

## Results

### Participant Characteristics

Of the 172 enrolled patients, 80.2% (n=138) were female; 75.6% (n=130) were White; 44.8% (n=77) identified as Hispanic, with 53.5% (n=92) selecting Spanish as their preferred language; 47.7% (n=82) had at least some college education; 45.3% (n=78) were unemployed, and 94.8% (n=163) had an estimated annual income below US $50,000 (Multimedia Appendix 2). Of those enrolled, 82 (47.7%) were classified as valid wear patients, providing 7566 valid wear days in total during the entire study period. Noncompliance with Fitbit wear was related to forgetfulness, rashes, and limited digital proficiency. Table 1 shows the baseline characteristics of valid wear patients whose activity data were analyzed in this work. Compared to those who did not meet the valid wear criteria (n=90, Multimedia Appendix 2), patients with valid wear were younger (mean 46.9, SD 13.0 vs mean 51.4, SD 9.6 years, *P*=.01), less likely to be Hispanic (37% vs 55%, *P*=.03) or Spanish-speaking (43% vs 63%, *P*=.01), and less likely to be married (26% vs 54%, *P*<.001). No other significant difference was observed in baseline characteristics and PROs between the valid wear and the invalid wear group.

**Table 1:** Characteristics and COVID experience of valid wear patients at baseline. Valid wear patients had at least 20 hours of valid Fitbit data per day for at least 7 days throughout this study.

Characteristics		Valid wear (n=82)
Age, mean (SD)		46.9 (13.0)
**Sex, n (%)**		
	Female	68 (83)
	Male	14 (17)
**Race, n (%)**		
	White	59 (72)
	Non-White	13 (16)
	Unknown	10 (12)
**Ethnicity, n (%)**		
	Non-Hispanic or Latino	50 (61)
	Hispanic	29 (35)
	Unknown	3 (4)
**Education, n (%)**		
	Grade 5 or less	6 (7)
	Grade 6 to 8	4 (5)
	Grade 9 to 12	25 (30)
	Some college or college	39 (48)
	Postgraduate	5 (6)
	Unknown	3 (4)
**Employment status, n (%)**		
	Employed	35 (43)
	Unemployed	35 (43)
	Student	5 (6)
	Retired	2 (2)
	Unknown	5 (6)
**Housing status, n (%)**		
	Rent	64 (78)
	Own	10 (12)
	Homeless	5 (6)
	Unknown	3 (4)
**Marital status, n (%)**		
	Single	40 (49)
	Married	21 (26)
	Other	21 (26)
**Estimated annual income (US $), n (%)**		
	0	25 (30)
	1-19,999	30 (37)
	20,000-49,999	22 (27)
	50,000+	2 (2)
	Unknown	3 (4)
**Preferred language, n (%)**		
	English	47 (57)
	Spanish	35 (43)
**Frequency of completing 150 minutes per week of moderate-intensity physical activity before contracting COVID-19, n (%)**		
	Never	19 (23)
	Very few weeks	13 (16)
	Some weeks	4 (5)
	Most weeks	11 (13)
	Every week	28 (34)
	Unknown	7 (9)
**Frequency of completing 150 minutes per week of vigorous-intensity physical activity before contracting COVID-19, n (%)**		
	Never	47 (57)
	Very few weeks	9 (11)
	Some weeks	5 (6)
	Most weeks	2 (2)
	Every week	13 (16)
	Unknown	6 (7)
Admitted to the hospital due to COVID-19, n (%)		17 (21)
Admitted to the ICU^a^ due to COVID-19, n (%)		9 (11)
Prescribed supplementary oxygen support due to COVID-19, n (%)		15 (18)
Intubated due to COVID, n (%)		2 (2)
**Long COVID and ME/CFS^b^ diagnosis, n (%)**		
	Long COVID only	71 (87)
	ME/CFS only	4 (5)
	Both long COVID and ME/CFS	6 (7)

^a^ICU: intensive care unit.

^b^ME/CFS: myalgic encephalomyelitis/chronic fatigue syndrome.

### Fitbit Findings and Patient Categorization

Fitbit data between baseline and 6-month dates were used for subsequent analyses in consideration of the number of patients contributing valid data and their potential association with PROs at 6-month follow-up. These include 4141 days of Fitbit data from valid wear patients, with each patient providing 50.5 (SD 38.5) days of data on average (median 42, IQR 20-70.75). The distribution of days of valid Fitbit data since Fitbit deployment through the 6-month PRO assessment for each valid wear participant can be found in Multimedia Appendix 3. Noncompliance with PRO completion was associated with survey length, difficulty reaching patients, and symptom-related challenges.

After applying MVPA categorization using average daily MVPA minutes between baseline and 6 months, valid wear patients (n=82) were classified into an MVPA-active subgroup (n=41) and an MVPA-inactive subgroup (n=41). MVPA-inactive patients had an average number of valid wear days of 60.2 (SD 41.9; median 51, IQR 23-87), whereas MVPA-active patients had an average of 40.8 (SD 32.4; median 28, IQR 16-63). The Wilcoxon rank test indicated that the MVPA-inactive group had significantly more valid wear days than the MVPA-active group (*P*=.33). Table 2 summarizes average daily Fitbit characteristics of the two groups. Consistent with their labels, the MVPA-active group had significantly higher MVPA minutes (*P*<.001), steps (*P*<.001), maximum HR (*P*<.001), and significantly lower percentages of sedentary time (*P*=.002) than the MVPA-inactive group.

**Table 2:** Average daily Fitbit characteristics of MVPA^a^-active and MVPA-inactive patients between baseline and 6 months. Patients were categorized by their individual average daily MVPA minutes.

Daily Fitbit characteristics	MVPA-active (n=41), mean (SD)	MVPA-inactive (n=41), mean (SD)	*P* value
Sedentary time/wear time (%)	66.8 (9.8)	74 (8.8)	.002
Lightly active minutes	283.2 (81.0)	242.1 (82.7)	.06
MVPA minutes	46.9 (31.4)	8.2 (6.3)	<.001
Steps	8966 (3818)	5744 (2582)	<.001
Resting heart rate	70.7 (9.0)	70.6 (8.7)	.79
Maximum heart rate	127.8 (8.5)	121.2 (8.1)	<.001
Go-to-bed time	23.99 (1.51)	24.11 (1.70)	.98
Get-up time	7.60 (1.57)	8.01 (1.94)	.37
Hours asleep	6.83 (1.36)	6.96 (1.04)	.91
Asleep time/time in bed (%)	87.4 (2.7)	87.1 (3.7)	.85

^a^MVPA: moderate to vigorous physical activity.

### Patient-Reported Outcomes

The 2 groups reported significantly different scores in the physical function, fatigue, pain interference, social roles, and dyspnea scales at baseline (Table 3). The differences in group means all reached the general meaningful change threshold of 3 T-score points for group comparisons [43]. After converting into severity categories (ie, acceptable or mild, moderate concern, or significant concern) using standardized cutoff scores of PROMIS scales, the differences in physical function, fatigue, and dyspnea scores remained statistically significant (*P* values were .03, .006, and .007, respectively), indicating that patients in the MVPA-inactive subgroup experienced more severe problems as reflected by the 3 subscales than those in the MVPA-active subgroup at baseline. Similar differences were observed in the severity scores of related symptoms.

**Table 3:** PROs^a^ of MVPA^b^-active and MVPA-inactive patients at baseline^c^.

PROs		MVPA-active (n=41)	MVPA-inactive (n=41)	*P* value
PHQ-2^d,e^ (0-6), ≥3^f^, n (%)		8 (20)	15 (37)	.08
PROMIS-29^g^ depression^e^ (20-80), mean (SD)		53 (10)	56 (11)	.21
GAD-7^e,h^ (0-21), mean (SD)		6.1 (5.0)	8.2 (5.7)	.12
PROMIS-29 anxiety^e^ (20-80), mean (SD)		56 (11)	57 (10)	.77
PROMIS-29 physical function^i^ (20-80), mean (SD)		38 (7)	34 (7)	.006
PROMIS-29 fatigue^e^ (20-80), mean (SD)		59 (10)	66 (8)	<.001
PROMIS-29 sleep disturbance^e^ (20-80), mean (SD)		58 (5)	58 (6)	.55
PROMIS-29 pain interference^e^ (20-80), mean (SD)		60 (10)	65 (9)	.03
PROMIS-29 social roles^i^ (20-80), mean (SD)		47 (11)	40 (10)	.02
PROMIS^j^ dyspnea^e^ (20-80), mean (SD)		56 (10)	63 (12)	.002
PROMIS cognition^i^ (20-80), mean (SD)		46 (12)	41 (10)	.06
**Fatigue, tiredness, or exhaustion**				
	Yes, n (%)	36 (88)	39 (95)	.43
	Severity score^k^ (0-16), mean (SD)	9.2 (4.2)	12.3 (4.2)	.001
**Muscle pain, muscle cramps, or muscle aches**				
	Yes, n (%)	33 (80)	32 (78)	.99
	Severity score^k^ (0-16), mean (SD)	7.0 (4.9)	8.1 (5.7)	.37
**Joint pain**				
	Yes, n (%)	25 (61)	29 (71)	.48
	Severity score^k^ (0-16), mean (SD)	5.6 (5.3)	7.5 (5.5)	.15
**Unrefreshing sleep**				
	Yes, n (%)	29 (71)	34 (83)	.30
	Severity score^k^ (0-16), mean (SD)	7.1 (6.0)	9.8 (5.3)	.06
**Problems getting to sleep, sleeping through the night, or waking up on time**				
	Yes, n (%)	34 (83)	37 (90)	.52
	Severity score^k^ (0-16), mean (SD)	7.1 (5.7)	10.4 (4.7)	.007
**Forgetfulness or memory problems**				
	Yes, n (%)	24 (59)	31 (76)	.16
	Severity score^k^ (0-16), mean (SD)	5.2 (5.6)	8.1 (5.5)	.03
**Difficulty thinking or concentrating**				
	Yes, n (%)	26 (63)	35 (85)	.04
	Severity score^k^ (0-16), mean (SD)	5.3 (5.2)	8.7 (5.4)	.006
**Dizziness or lightheadedness**				
	Yes, n (%)	29 (71)	33 (80)	.44
	Severity score^k^ (0-16), mean (SD)	4.8 (4.5)	4.4 (3.6)	.98
Post-exertional malaise^l^, yes, n (%)		32 (78)	30 (73)	.80

^a^PRO: patient-reported outcome.

^b^MVPA: moderate to vigorous physical activity.

^c^Ranges of patient-reported outcome scores are indicated in parentheses. PROMIS T-scores have a mean of 50 and an SD of 10 in the reference population and typically fall between 20 and 80.

^d^PHQ-2: Patient Health Questionnaire-2.

^e^On PHQ-2, GAD-7, and all PROMIS subscales except physical function, social roles, and cognition, a higher score indicates more severe problems or concerns.

^f^A PHQ-2 score of 3 or greater indicates that major depressive disorder is likely.

^g^PROMIS-29: Patient-Reported Outcomes Measurement Information System-29.

^h^GAD-7: Generalized Anxiety Disorder-7.

^i^On PROMIS physical function, social roles, and cognition subscales, a lower score indicates more severe problems or concerns.

^j^PROMIS: Patient-Reported Outcomes Measurement Information System.

^k^Symptom severity scores were calculated using the scoring algorithm of the CDC Symptom Inventory [34,35]. A higher score indicates that the symptom is more frequent or more intense. A score of 0 means no symptom.

^l^Post-exertional malaise was measured by a yes, no, or do not know question asking whether any of the symptoms get worse for at least 24 hours after activity.

Table 4 provides average individual changes in PROs from baseline to 3 months and 6 months, showing the direction and magnitude of the trends in PROs. However, the effect of other factors is not considered, and the sample size is limited due to the pairwise within-subject calculation. To address this, we further analyzed longitudinal associations between MVPA levels and PROs with LME regression, which is able to handle missing values while modeling the effects of multiple factors.

**Table 4:** Average individual changes in PROs^a^ from baseline to 3 and 6 months^b^.

PRO change scores	From baseline to 3 months		From baseline to 6 months	
	MVPA^c^-active	MVPA-inactive	MVPA-active	MVPA-inactive
PHQ-2^d,e^ (0-6), mean (SD)	+0.2 (1.7)	–0.3 (1.8)	+0.2 (1.4)	–0.8 (2.0)
PROMIS-29^f^ depression^e^ (20-80), mean (SD)	+1 (9)	0 (9)	+2 (8)	–2 (9)
GAD-7^e,g^ (0-21), mean (SD)	+0.8 (5.2)	–0.7 (4.6)	+1.4 (4.6)	–1.6 (6.0)
PROMIS-29 anxiety^e^ (20-80), mean (SD)	–4 (11)	–1 (12)	–1 (6)	–2 (11)
PROMIS-29 physical function^h^ (20-80), mean (SD)	–1 (6)	+1 (6)	–1 (8)	+2 (7)
PROMIS-29 fatigue^e^ (20-80), mean (SD)	0 (9)	–2 (9)	+2 (10)	–3 (9)
PROMIS-29 sleep disturbance^e^ (20-80), mean (SD)	–1 (6)	–1 (7)	0 (8)	–2 (7)
PROMIS-29 pain interference^e^ (20-80), mean (SD)	+1 (10)	–1 (11)	0 (10)	–2 (9)
PROMIS-29 social roles^h^ (20-80), mean (SD)	0 (11)	+3 (10)	0 (11)	+8 (10)
PROMIS dyspnea^e^ (20-80), mean (SD)	+1 (12)	–4 (13)	–1 (10)	–2 (13)
PROMIS cognition^h^ (20-80), mean (SD)	–1 (9)	+2 (9)	0 (13)	+4 (9)

^a^PRO: patient-reported outcome.

^b^Ranges of PRO scores are indicated in parentheses. PROMIS T-scores have a mean of 50 and an SD of 10 in the reference population and typically fall between 20 and 80. Data from symptom severity scores is not included due to limited sample sizes, since only those who have reported a symptom would receive follow-up questions about that symptom in the next round of questionnaires.

^c^MVPA: moderate to vigorous physical activity.

^d^PHQ-2: Patient Health Questionnaire-2.

^e^On PHQ-2, GAD-7, and all PROMIS subscales except physical function, social roles, and cognition, a higher score indicates more severe problems or concerns.

^f^PROMIS-29: Patient-Reported Outcomes Measurement Information System-29.

^g^GAD-7: Generalized Anxiety Disorder-7.

^h^On PROMIS physical function, social roles, and cognition subscales, a lower score indicates more severe problems or concerns.

### Longitudinal Associations Between MVPA and PROs

LME analysis indicated a statistically significant effect of MVPA level on scores of several PROs over time. For every 3 months of being MVPA-active compared with MVPA-inactive, the coefficient estimates are –1.94 (95% CI –3.05 to –0.82, *P*<.001) for raw scores of PROMIS-29 ability to participate in social roles and activities scale (abbreviated as PROMIS-29 social roles) and –4.21 (95% CI –6.64 to –1.78, *P*<.001) for its T-scores. The latter is greater than both the general meaningful change threshold of 3 T-score points for group comparison on PROMIS scales and the minimal important change values of 0.4-2.2 T-score points estimated by previous studies for the PROMIS-29 social roles scale [43,44]. For raw physical function scores, this coefficient is –1.00 (95% CI –1.97 to –0.04, *P*=.04) but is no longer significant when regressing on T-scores (estimate=–1.35, 95% CI –3.00 to 0.30, *P*=.11). Considering that a lower score indicates more severe problems on both physical function and social roles subscales, these negative coefficient estimates suggest a potential negative effect of high MVPA levels or a beneficial effect of low MVPA levels over time. Aligning with LME regression results, average individual change scores (Table 4) on physical function and social roles scales show that the scores of MVPA-active patients remained around the same on average, while patients in the MVPA-inactive group had increased scores on average. Together, these findings indicate that MVPA-inactive patients experienced more improvements in outcomes on average after 6 months compared to MVPA-active patients, especially in the ability to participate in social roles.

LME regression of long COVID symptom severity scores identified a statistically significant effect of MVPA group over time on symptoms related to sleep quality (ie, problems getting to sleep, sleeping through the night, or waking up on time), with a coefficient estimate of 2.06 (95% CI 0.40 to 3.71, *P*=.02) for being MVPA-active over 3 months. Similar to the observations from PROMIS-29 scores, this positive estimate suggests that MVPA-inactive patients had more improvements in sleep quality after 6 months than MVPA-active patients. However, this effect was not reflected by the PROMIS-29 sleep disturbance scale.

The effect of MVPA level over time is not significant for any other PROs. In addition, the main effects of MVPA groups at baseline in LME regression are consistent with baseline comparisons in Table 3 when baseline PRO scores are not included as a predictor. The addition of other Fitbit or demographic variables did not result in improved model fitness. The only new variable that appeared to be statistically significant was step count, which is highly correlated with MVPA minutes. Model diagnostic tests indicated that the fitted models were valid. Subsequent sensitivity analysis using slightly different thresholds for the classification of MVPA groups and data inclusion in LME regression produced the same findings, with only small fluctuations in coefficient estimates and *P* values. For example, 2 patients had average daily MVPA levels near the classification cutoff. After moving them to the other group, the coefficient estimate of MVPA-time interaction remained significant. Removal of patients with near-cutoff numbers of valid wear days and overly skewed or scattered valid wear days (eg, patient 039, 064, and 137) produced the same findings. We also tried removing 9 patients who had valid PRO scores at only one time point, and findings remained the same. PRO data from patients who missed 1 time point were considered valid and included in LME models.

### Exploratory Analyses

The GMM of day-level Fitbit time series identified 1 cluster of resting HR, 1 cluster of step counts, and 2 clusters of MVPA minutes. The GMM model of daily resting HR has an intercept of 71.5 (*P*<.001) beats per minute, and a slope of –0.023 (*P*=.01) beats per minute per day, indicating a slight decreasing trend in resting HR over time on average. The intercept represents the modeled average value on day 0 of Fitbit deployment under specified model settings and model constraints. The step count model has an intercept of 7289 steps (*P*<.001) and a slope of 2.51 (*P*=.53) steps per day, indicating that the average step count of the patients was stable over time in general. The 2 clusters of daily MVPA minutes have sizes of 70 and 12 patients, intercepts of 18.4 and 68.2 minutes (both with *P*<.001), and slopes of –0.029 (*P*=.46) and 0.20 (*P*=.39) minutes per day, respectively. This indicates that there were an MVPA-active subgroup and an MVPA-inactive subgroup, but the activities of both subgroups did not exhibit a clear trend over time. As GMM does not provide specific cutoffs for patient clusters and GMM-identified clusters may be of insufficient sample size, we did not use GMM-identified clusters for further analysis, but instead selected the WHO guideline, which is more reusable and comparable for future studies. Due to concerns over the missingness and quality of the sleep data from Fitbit, we did not analyze this data.

Further analyses were conducted to study potential factors related to the observed MVPA levels. Employment status at baseline was similar between MVPA-active and MVPA-inactive groups, and differences in Fitbit measures remained significant after adjusting for employment in a 2-way ANOVA, indicating that MVPA differences were not explained by employment status alone. Multivariate linear regression of MVPA minutes against demographic characteristics and baseline survey scores, with either a logistic model or a 0-inflated negative binomial model, revealed sex and baseline PROMIS-29 fatigue score as significant predictors. Specifically, males and individuals with less severe fatigue at baseline were correlated with being MVPA-active during this study’s period.

Other exploratory analyses on the Fitbit data, including dimensionality reduction of average Fitbit features (to study potential patient clusters), time series clustering of minute-level data (to study daily activity patterns), weekday-to-weekend and weekly comparisons (to study weekly patterns), and alignment of patient activities on adjacent days (to study day-to-day variations or potential activity pacing behaviors), did not produce meaningful findings beyond reported results and existing literature.

## Discussion

### Principal Findings

This study collected Fitbit-measured physical activity data and PROs from a subset of long COVID and ME/CFS patients in the LC&FIRP study to identify longitudinal patterns and examine the overlay of PROs in relation to patient characteristics. The results showed that patients could remain MVPA-active despite experiencing symptoms. According to the Physical Activity Guidelines for Americans, moderate housework or yardwork, such as carrying groceries or raking the yard, may reach the threshold of moderate activity [45]. One of the main characteristics of long COVID is the impact of symptoms that can alter functionality within these critical everyday tasks [46]. While we do not have records of why or how some patients maintained high activity levels, one factor to consider is the socioeconomic status of our patient cohort. FHCSD is one of the ten largest FQHCs in the nation, and the vast majority of patients are low-income [47]. It is important to analyze data across diverse patient populations, as those at an FQHC may have experienced household or financial demands, including the need to continue working. Further study of employment type and partner’s employment may provide additional insight. These factors highlight both the need for pacing, planning, and prioritizing as well as the real challenges that prevent their implementation [48].

In long COVID, where fatigue and PEM are common, physical activity should be recommended with care in an individualized approach. In our study, 75.6% (62/82) of patients reported PEM, and the proportions in the 2 MVPA subgroups were not significantly different (*P*=.80). The high rate of PEM may reflect our patient population’s responsibilities when it comes to providing and caring for their families. Although some studies have demonstrated improvements in metrics related to physical function after physical rehabilitation programs, few addressed PEM, and those that did found no improvement in exercise performance following structured exercise interventions [49-54].

In our attempts to quantify PEM, we found that it was difficult to establish parameters due to the diversity in patient experiences. Although symptoms of PEM vary, fatigue, cognitive dysfunction, and sleep problems are reported with high frequency [55]. Velez-Santamaria et al [46] examined the changes in fatigue severity in long COVID participants, finding that it worsened significantly, with 92.4% of participants meeting the criteria for ME/CFS, while Stussman et al [56] highlighted the diverse onset, duration, and recovery patterns of PEM symptoms. Similar to our study, this heterogeneity underscores the need to tailor treatment strategies based on the severity of fatigue, symptoms, and the patient’s lifestyle [56].

Of the 11 published studies on long COVID and wearable devices, ours was the only one to use PROMIS-29 as the primary PRO measure [26]. We found that at baseline, MVPA-inactive patients experienced more severe problems with physical function, fatigue, dyspnea, and sleep quality than MVPA-active patients. On average, after 6 months, MVPA-inactive patients reported some recovery in the domains of sleep quality and the ability to participate in social roles, compared to MVPA-active patients whose metrics worsened or remained around the same. Other studies used different PROS, including the Fatigue Assessment Scale [49], Short Form 36 Health Survey [50], or the COVID-19 Yorkshire Rehabilitation Scale [57], making comparisons challenging due to heterogeneity in scales, symptom emphasis, and reference periods. As PROMIS-29 was not used in prior studies, direct trajectory comparisons with other cohorts are limited.

Previous studies also examined the association between physical activity and patient with long COVID’s experiences. Humphreys et al [58] conducted qualitative interviews with patients with long COVID, with one of the main themes being the struggle with impaired physical function. In a cross-sectional study by Wright et al [59], about 75% of the participants reported that physical activity worsened their long COVID symptoms. A later cross-sectional study by Vélez-Santamaría et al [46], involving multiple quantitative questionnaires similar to our study, revealed a significant association between impaired functionality, lower physical activity levels, and worsened quality of life in patients with long COVID. Rekeland et al [60] also reported similar associations between physical activity, ME/CFS severity, and PROs in patients with ME/CFS. Findings in our study are consistent with and complementary to those studies. To our knowledge, our study is the first to report longitudinal patterns of physical activity in lower-income patients with long COVID using a wearable device and PROs. Other studies exist, but do not focus on similar cohorts [61-63].

Our finding that a lower MVPA level was associated with more improvements in outcomes in the long-term seems to suggest some benefit from reduced activity. Given our observational results, we propose that it may be beneficial for health professionals to ask patients about their normal pattern of physical activity and symptoms that follow or worsen after activity. If the patient’s responsibilities require a substantial amount of MVPA, strategies for mitigating its potentially negative effects on symptoms could be explored. One potential strategy is activity pacing, that is, dividing physical activities into multiple portions that are more manageable and balancing them with rests [64]. In our study, we did not observe clear indications in the exploratory analysis (result not shown) that patients paced their activities on the day level because they maintained a relatively stable amount of MVPA on weekdays over time. In addition, our PROs were only conducted every 3 months, which may have resulted in difficulty for the patient to recall certain activities that caused PEM. Due to the exploratory nature of our study, results should be interpreted as hypothesis-generating.

The findings of this study should be interpreted in light of its limitations. First, the sample size of this study was relatively small (n=82 for valid wear patients) and was observational in nature, which limits the extent to which results can be generalized. Second, exploratory analysis is typically used to generate hypotheses of effects and associations rather than to confirm them definitively. The goal is often to identify potential patterns, associations, or areas for further study, rather than to make conclusions about the data. It is important to interpret the *P* values reported with caution, given that the number of statistical tests performed increases the risk of type I errors (false positives). As such, our findings provide insights into the longitudinal trajectory of patients with long COVID and should be used to guide future research. Third, due to concerns over the missingness and quality of Fitbit data on sleep and HRV, we were unable to investigate these characteristics. Future studies could consider collecting these measures through other wearable devices or even including biological markers to better understand these behavioral and physiological aspects among patients with long COVID and related IACCs. Fourth, as a substudy of a large intervention program, this study did not contain a healthy control group or other illness comparison groups, unlike other studies [62,63]. Therefore, patients were compared to themselves in the past or to other patients with different characteristics. Fifth, the applicability of the WHO MVPA guideline to patients with long COVID’s needs further validation. Lastly, our sample was focused on patients at a single site, which may affect generalizability to broader populations, whereas other studies had a more diverse population [26].

Considering that repeated measures studies could be undermined by the regression to the mean phenomenon, we conducted baseline-adjusted LME regression analysis, which appropriately accounts for patient-level variability in repeated measures. Moreover, the major effect we observed on the PROMIS-29 social roles scale is greater than the meaningful change thresholds of the PROMIS scales, which is unlikely to be solely due to regression to the mean. Nevertheless, a large, real-world interventional cohort that includes a diverse set of patients may help phenotype patients in a more comprehensive manner, potentially leading to a better understanding of the disease, treatment strategies, and the development of effective interventions. Lastly, our project lacked the ability to monitor and follow up with patients in real time when they were experiencing a worsening symptom or PEM. The use of ecological momentary assessment is a crucial next step to better comprehend the sporadic nature of symptoms and PEM in patients with the goal of gathering information about daily patterns of symptoms to identify triggers for the worsening of symptoms [65].

### Conclusions

In conclusion, our results highlight the complex nature and diverse impact of long COVID and ME/CFS, drawing attention to the value of combining self-reported symptoms and objective physical activity data when evaluating individuals. Furthermore, the results suggest that disease experience is individualized. As this study was exploratory in nature, our findings could help lay the groundwork for clinician-patient interactions and for tailoring rehabilitation efforts and guiding future research. Our paper highlights the importance of continuing research in the field of long COVID and ME/CFS. Next steps could include a deeper dive into physiological aspects such as resting metabolic rate, ecological momentary assessment, HRV, and sleep over a continuous period. Using a combination of different wearables in a larger sample size could help to further the understanding of personal trajectories of long COVID experience, hopefully leading to personalized treatment options and better tracking of disease progression.

## Appendix

Multimedia Appendix 1 STROBE checklist for cohort studies.

Multimedia Appendix 2 Complete patient characteristics and PROs at baseline. PRO: patient reported outcomes.

Multimedia Appendix 3 Distributions of valid wear days of each valid wear patient between Fitbit deployment and 6-month PRO assessment. Each point marks a valid wear day. Patients are colored by their MVPA group. The x-axis does not correspond to survey dates. MVPA: moderate to vigorous physical activity; PRO: patient reported outcomes.

## Abbreviations

CDC: Centers for Disease Control and Prevention
FHCSD: Family Health Centers of San Diego
FQHC: Federally Qualified Health Center
GAD-7: Generalized Anxiety Disorder-7
GMM: growth mixture modeling
HR: heart rate
HRV: heart rate variability
IACC: infection-associated chronic condition
LC&FIRP: Long COVID and Fatiguing Illness Recovery Program
LME: linear mixed effect
ME/CFS: myalgic encephalomyelitis/chronic fatigue syndrome
MET: metabolic equivalent
MVPA: moderate to vigorous physical activity
PEM: post-exertional malaise
PHQ-2: Patient Health Questionnaire-2
PRO: patient-reported outcome
PROMIS: Patient-Reported Outcomes Measurement Information System
PROMIS-29: Patient-Reported Outcomes Measurement Information System-29
STROBE: Strengthening the Reporting of Observational Studies in Epidemiology
teleECHO: Extension for Community Healthcare Outcomes
WHO: World Health Organization

## Data Availability

The deidentified original datasets used in this study are available from the corresponding author upon reasonable request. Due to ethical, legal, and privacy considerations, we refrain from sharing the original data through a public repository. Necessary summary data for study findings have been provided in the main text or as supplementary materials.
