# Correction: Characterization of Post-Viral Infection Behaviors Among Patients With Long COVID: Prospective, Observational, Longitudinal Cohort Analyses of Fitbit Data and Patient-Reported Outcomes

**DOI:** 10.2196/92848

**Published:** 2026-02-18

**Authors:** Tianmai M Zhang, Sydney P Sharp, John D Scott, Douglas Taren, Jane C Samaniego, Elizabeth R Unger, Jeanne Bertolli, Jin-Mann S Lin, Christian B Ramers, Job G Godino

**Affiliations:** 1 Laura Rodriguez Research Institute Family Health Centers of San Diego San Diego, CA United States; 2 Department of Biomedical Informatics and Medical Education University of Washington Seattle, WA United States; 3 Department of Medicine University of Washington Seattle, WA United States; 4 Department of Pediatrics Nutrition Section University of Colorado Aurora, CO United States; 5 National Center for Emerging and Zoonotic Infectious Diseases Centers for Disease Control and Prevention Atlanta, GA United States; 6 School of Medicine University of California, San Diego La Jolla, CA United States; 7 School of Public Health San Diego State University San Diego, CA United States; 8 Global Hepatitis Program Clinton Health Access Initiative Boston, MA United States; 9 Herbert Wertheim School of Public Health and Human Longevity Science University of California, San Diego La Jolla, CA United States; 10 Exercise and Physical Activity Resource Center University of California, San Diego La Jolla, CA United States

In “Characterization of Post-Viral Infection Behaviors Among Patients With Long COVID: Prospective, Observational, Longitudinal Cohort Analyses of Fitbit Data and Patient-Reported Outcomes”, the authors made one correction.

In the abstract, the sentence on longitudinal results has been amended to replace “MVPA-inactive” with “MVPA-active” and “decreased” with “statistically less improvements.”

In the abstract, the following sentence was revised:

Longitudinal analysis found that MVPA-inactive patients showed a decreased ability to participate in social roles (estimated group difference=–4.21 T-score points over 3 months, 95% CI –6.64 to –1.78, P<.001) and a higher intensity of sleep symptoms (estimated group difference=2.06 severity score points over 3 months, 95% CI 0.40 to 3.71, P=.02) over time.

The sentence now reads:

*Longitudinal analysis found that MVPA-active patients had statistically less improvements in the ability to participate in social roles (estimated group difference=–4.21 T-score points over 3 months, 95% CI –6.64 to –1.78, P<.001) and the intensity of sleep symptoms (estimated group difference=2.06 severity score points over 3 months, 95% CI 0.40 to 3.71, P=.02) over time.*

The correction will appear in the online version of the paper on the JMIR Publications website, together with the publication of this correction notice. Because this was made after submission to PubMed, PubMed Central, and other full-text repositories, the corrected article has also been resubmitted to those repositories.

