# Supplementary material for: Characterization of Post-Viral Infection Behaviors Among Patients With Long COVID: Prospective, Observational, Longitudinal Cohort Analyses of Fitbit Data and Patient-Reported Outcomes
Source: JMIR Form Res. 2025 Dec 31;9:e77644. doi: 10.2196/77644 (PMC12805324; doi:10.2196/77644)
Supplement: Multimedia Appendix 2 [file formative_v9i1e77644_app2.docx]

**Table S1.** Patient characteristics and patient reported outcomes (PROs) at baseline.

| **Demographics** | **All**  N=172 | **Valid Wear**  n=82 | **Invalid Wear**  n=90 | ***P*** |
| --- | --- | --- | --- | --- |
| Age  Mean (SD) | n=172  49.3 (11.6) | n=82  46.9 (13.0) | n=90  51.4 (9.6) | .011 |
| Sex, n (%)  Female  Male | n=172  138 (80%)  34 (20%) | n=82  68 (83%)  14 (17%) | n=90  70 (78%)  20 (22%) | .51 |
| Race  White  Others | n=148  130 (88%)  18 (12%) | n=72  59 (82%)  13 (18%) | n=76  71 (93%)  5 (7%) | .06 |
| Ethnicity  Non-Hispanic or Latino  Hispanic | n=166  89 (54%)  77 (46%) | n=79  50 (63%)  29 (37%) | n=87  39 (45%)  48 (55%) | .026 |
| Education  Grade 5 or less  Grade 6-8  Grade 9-12  Some college or college  Postgraduate | n=162  10 (6%)  12 (7%)  58 (36%)  73 (45%)  9 (6%) | n=79  6 (8%)  4 (5%)  25 (32%)  39 (49%)  5 (6%) | n=83  4 (5%)  8 (10%)  33 (40%)  34 (41%)  4 (5%) | .53 |
| Employment status  Employed  Unemployed  Student  Retired | n=152  65 (43%)  78 (51%)  6 (4%)  3 (2%) | n=77  35 (45%)  35 (45%)  5 (6%)  2 (3%) | n=75  30 (40%)  43 (57%)  1 (1%)  1 (1%) | .24 |
| Has contracting COVID-19 impacted your employment status?  Yes | n=167  128 (77%) | n=78  58 (74%) | n=89  70 (79%) | .64 |
| Housing status  Rent  Own  Homeless | n=165  125 (76%)  26 (16%)  14 (8%) | n=79  64 (81%)  10 (13%)  5 (6%) | n=86  61 (71%)  16 (19%)  9 (10%) | .32 |
| Marital status  Single  Married  Others | n=168  59 (35%)  68 (40%)  41 (24%) | n=81  40 (49%)  21 (26%)  20 (25%) | n=87  19 (22%)  47 (54%)  21 (24%) | <.001 |
| Income Annual (est.)  Mean (SD)  0  1-19,999  20,000-49,999  50,000+ | n=168  15.2k (14.6k)  45 (27%)  75 (45%)  43 (26%)  5 (3%) | n=79  14.1k (14.3k)  25 (32%)  30 (38%)  22 (28%)  2 (3%) | n=89  16.2k (14.8k)  20 (22%)  45 (51%)  21 (24%)  3 (3%) | .28  .36 |
| Language preferred  English  Spanish | n=172  80 (47%)  92 (53%) | n=82  47 (57%)  35 (43%) | n=90  33 (37%)  57 (63%) | .010 |
| **COVID Initial Experience** | **All**  N=172 | **Valid Wear**  n=82 | **Invalid Wear**  n=90 | ***P*** |
| Prior to contracting COVID-19, how frequently did you complete 150-minutes per week of moderate-intensity physical activity?  Never  Very few weeks  Some weeks  Most weeks  Every week | n=163  53 (33%)  35 (21%)  10 (6%)  17 (10%)  48 (29%) | n=75  19 (25%)  13 (17%)  4 (5%)  11 (15%)  28 (37%) | n=88  34 (39%)  22 (25%)  6 (7%)  6 (7%)  20 (23%) | .07 |
| Prior to contracting COVID-19, how frequently did you complete 150-minutes per week of vigorous-intensity physical activity?  Never  Very few weeks  Some weeks  Most weeks  Every week | n=165  111 (67%)  19 (12%)  11 (7%)  3 (2%)  21 (13%) | n=76  47 (62%)  9 (12%)  5 (7%)  2 (3%)  13 (17%) | n=89  64 (72%)  10 (11%)  6 (7%)  1 (1%)  8 (9%) | .51 |
| Were you admitted to the hospital due to COVID-19?  Yes | n=169  42 (25%) | n=79  17 (22%) | n=90  25 (28%) | .45 |
| Were you admitted to the ICU?  Yes | n=167  25 (15%) | n=78  9 (12%) | n=89  16 (18%) | .34 |
| Were you prescribed supplementary oxygen support?  Yes | n=167  37 (22%) | n=78  15 (19%) | n=89  22 (28%) | .51 |
| Were you intubated?  Yes | n=164  9 (5%) | n=78  2 (3%) | n=86  7 (8%) | / |
| **Medical History** | **All**  N=172 | **Valid Wear**  n=82 | **Invalid Wear**  n=90 | ***P*** |
| Long COVID and ME/CFS  Long COVID only  ME/CFS only  Both Long COVID and ME/CFS | n=172  156 (91%)  6 (3%)  9 (5%) | n=82  71 (87%)  4 (5%)  6 (7%) | n=90  85 (94%)  2 (2%)  3 (3%) | .29 |
| Obesity  Yes | n=168  70 (42%) | n=79  35 (44%) | n=89  35 (39%) | .62 |
| Fibromyalgia  Yes | n=168  17 (10%) | n=79  10 (13%) | n=89  7 (8%) | .44 |
| ME/CFS  Yes | n=168  19 (11%) | n=79  9 (11%) | n=89  10 (11%) | .99 |
| Spinal Cord Injury  Yes | n=168  8 (5%) | n=79  3 (4%) | n=89  5 (6%) | / |
| Cancer  Yes | n=167  6 (4%) | n=78  3 (4%) | n=89  3 (3%) | / |
| Anxiety  Yes | n=168  86 (51%) | n=79  45 (57%) | n=89  41 (46%) | .21 |
| Depression  Yes | n=168  74 (44%) | n=79  43 (54%) | n=89  31 (35%) | .016 |
| Chronic Lyme Disease  Yes | n=168  2 (1%) | n=79  2 (3%) | n=89  0 (0%) | / |
| Hypertension  Yes | n=168  49 (29%) | n=79  18 (23%) | n=89  31 (35%) | .12 |
| Other Cardiovascular Disease  Yes | n=168  18 (11%) | n=79  8 (10%) | n=89  10 (11%) | .99 |
| Diabetes  Yes | n=167  42 (25%) | n=78  18 (23%) | n=89  24 (27%) | .85 |
| Chronic Kidney Disease  Yes | n=167  7 (4%) | n=79  5 (6%) | n=88  2 (2%) | / |
| Chronic Liver Disease  Yes | n=168  7 (4%) | n=79  4 (5%) | n=89  3 (3%) | / |
| Asthma  Yes | n=168  37 (22%) | n=79  22 (28%) | n=89  15 (17%) | .13 |
| COPD  Yes | n=168  3 (2%) | n=79  1 (1%) | n=89  2 (2%) | / |
| Other Chronic Lung Disease  Yes | n=168  5 (3%) | n=79  3 (4%) | n=89  2 (2%) | / |
| Hyperthyroidism  Yes | n=168  6 (4%) | n=79  4 (5%) | n=89  2 (2%) | / |
| Stroke  Yes | n=167  2 (1%) | n=79  1 (1%) | n=88  1 (1%) | / |
| Other Neurological Disease  Yes | n=168  6 (4%) | n=79  5 (6%) | n=89  1 (1%) | / |
| Seasonal Allergies  Yes | n=168  68 (40%) | n=79  30 (38%) | n=89  38 (43%) | .64 |
| Immunodeficiency  Yes | n=168  12 (7%) | n=79  8 (10%) | n=89  4 (4%) | / |
| Anemia  Yes | n=168  33 (20%) | n=79  17 (22%) | n=89  16 (18%) | .70 |
| Substance Use Disorder  Yes | n=168  6 (4%) | n=79  4 (5%) | n=89  2 (2%) | / |
| Other Psychiatric Disease  Yes | n=168  10 (6%) | n=79  6 (8%) | n=89  4 (4%) | / |
| Eating Disorder  Yes | n=168  11 (7%) | n=79  5 (6%) | n=89  6 (7%) | .99 |
| IBD/IBS  Yes | n=168  21 (12%) | n=79  12 (15%) | n=89  9 (10%) | .45 |
| Sleep Disorders  Yes | n=166  45 (27%) | n=78  21 (27%) | n=88  24 (27%) | .99 |
| **Questionnaires**^a^ | **All**  N=172 | **Valid Wear**  n=82 | **Invalid Wear**  n=90 | ***P*** |
| PHQ-2^b^, [0-6]  >= 3^d^, n (%) | n=168  48 (29%) | n=78  23 (29%) | n=90  25 (28%) | .94 |
| PROMIS-29 Depression^b^, [20-80]  Mean (SD) | n=168  54 (10) | n=79  55 (10) | n=89  53 (10) | .17 |
| GAD-7^b^, [0-21]  Mean (SD) | n=167  6.8 (5.5) | n=78  7.2 (5.4) | n=89  6.5 (5.6) | .33 |
| PROMIS-29 Anxiety^b^, [20-80]  Mean (SD) | n=168  57 (11) | n=79  57 (10) | n=89  57 (11) | .97 |
| PROMIS-29 Physical Function^c^, [20-80]  Mean (SD) | n=168  36 (8) | n=79  36 (7) | n=89  35 (8) | .45 |
| PROMIS-29 Fatigue^b^, [20-80]  Mean (SD) | n=168  62 (10) | n=79  63 (10) | n=89  62 (10) | .49 |
| PROMIS-29 Sleep Disturbance^b^, [20-80]  Mean (SD) | n=168  57 (5) | n=79  58 (5) | n=89  57 (5) | .11 |
| PROMIS-29 Pain Interference^b^, [20-80]  Mean (SD) | n=168  62 (9) | n=79  63 (10) | n=89  62 (9) | .38 |
| PROMIS-29 Social Roles^c^, [20-80]  Mean (SD) | n=167  44 (11) | n=78  43 (11) | n=89  45 (10) | .42 |
| PROMIS Dyspnea^b^, [20-80]  Mean (SD) | n=166  59 (11) | n=78  60 (12) | n=88  59 (11) | .49 |
| PROMIS Cognition^c^, [20-80]  Mean (SD) | n=166  44 (12) | n=78  43 (12) | n=88  44 (11) | .54 |
| **Symptoms**^e^ | **All**  N=172 | **Valid Wear**  n=82 | **Invalid Wear**  n=90 | ***P*** |
| Fatigue, Tiredness, or Exhaustion  Yes, n (%)  Severity score, [0-16], mean (SD) | n=166  160 (96%)  10.7 (4.6) | n=79  75 (94%)  10.8 (4.5) | n=87  85 (98%)  10.6 (4.8) | /  .99 |
| Muscle Pain, Muscle Cramps, or Muscle Aches  Yes, n (%)  Severity score, [0-16], mean (SD) | n=168  135 (80%)  7.5 (5.3) | n=79  65 (82%)  7.5 (5.3) | n=89  70 (79%)  7.4 (5.3) | .69  .96 |
| Joint Pain  Yes, n (%)  Severity score, [0-16], mean (SD) | n=168  125 (74%)  7.3 (5.5) | n=79  54 (68%)  6.6 (5.5) | n=89  71 (80%)  8.0 (5.5) | .13  .06 |
| Unrefreshing sleep  Yes, n (%)  Severity score, [0-16], mean (SD) | n=167  128 (77%)  7.9 (5.7) | n=78  63 (81%)  8.4 (5.8) | n=89  65 (73%)  7.4 (5.7) | .32  .24 |
| Problems getting to sleep, sleeping through the night, or waking up on time  Yes, n (%)  Severity score, [0-16], mean (SD) | n=168  137 (82%)  8.1 (5.6) | n=79  71 (90%)  8.8 (5.4) | n=89  66 (74%)  7.5 (5.7) | .015  .16 |
| Forgetfulness/memory problems  Yes, n (%)  Severity score, [0-16], mean (SD) | n=167  127 (76%)  7.1 (5.7) | n=78  55 (71%)  6.7 (5.7) | n=89  72 (81%)  7.4 (5.6) | .17  .40 |
| Difficulty thinking or concentrating  Yes, n (%)  Severity score, [0-16], mean (SD) | n=167  133 (80%)  7.4 (5.6) | n=78  61 (78%)  7.1 (5.5) | n=89  72 (81%)  7.7 (5.7) | .81  .51 |
| Dizziness or Lightheadedness  Yes, n (%)  Severity score, [0-16], mean (SD) | n=166  129 (78%)  5.0 (4.4) | n=79  62 (78%)  4.6 (4.1) | n=87  67 (77%)  5.4 (4.7) | .97  .34 |
| Post-exertional malaise^f^  Yes, n (%) | n=146  125 (86%) | n=68  62 (91%) | n=78  63 (81%) | .12 |

^a^Ranges of PRO scores are indicated in square brackets. PROMIS T-scores have a mean of 50 and standard deviation of 10 in reference population and typically fall between 20 and 80.

^b^On PHQ-2, GAD-7, and all PROMIS subscales except Physical Function, Social Roles, and Cognition, a higher score indicates more severe problems or concerns.

^c^On PROMIS Physical Function, Social Roles, and Applied Cognition subscales, a lower score indicates more severe problems or concerns.

^d^A PHQ-2 score of 3 or greater indicates that major depressive disorder is likely.

^e^Symptom severity scores were calculated using the scoring algorithm of the CDC Symptom Inventory.^41,42^ A higher score indicates that the symptom is more frequent and/or more intense. A score of 0 means no symptom.

^f^Post-exertional malaise was measured by a yes/no/don’t know question asking whether any of the symptoms get worse for at least 24 hours after activity.
