# Supplementary figures and images for: Characterization of Post-Viral Infection Behaviors Among Patients With Long COVID: Prospective, Observational, Longitudinal Cohort Analyses of Fitbit Data and Patient-Reported Outcomes
Source: JMIR Form Res. 2025 Dec 31;9:e77644. doi: 10.2196/77644 (PMC12805324; doi:10.2196/77644)

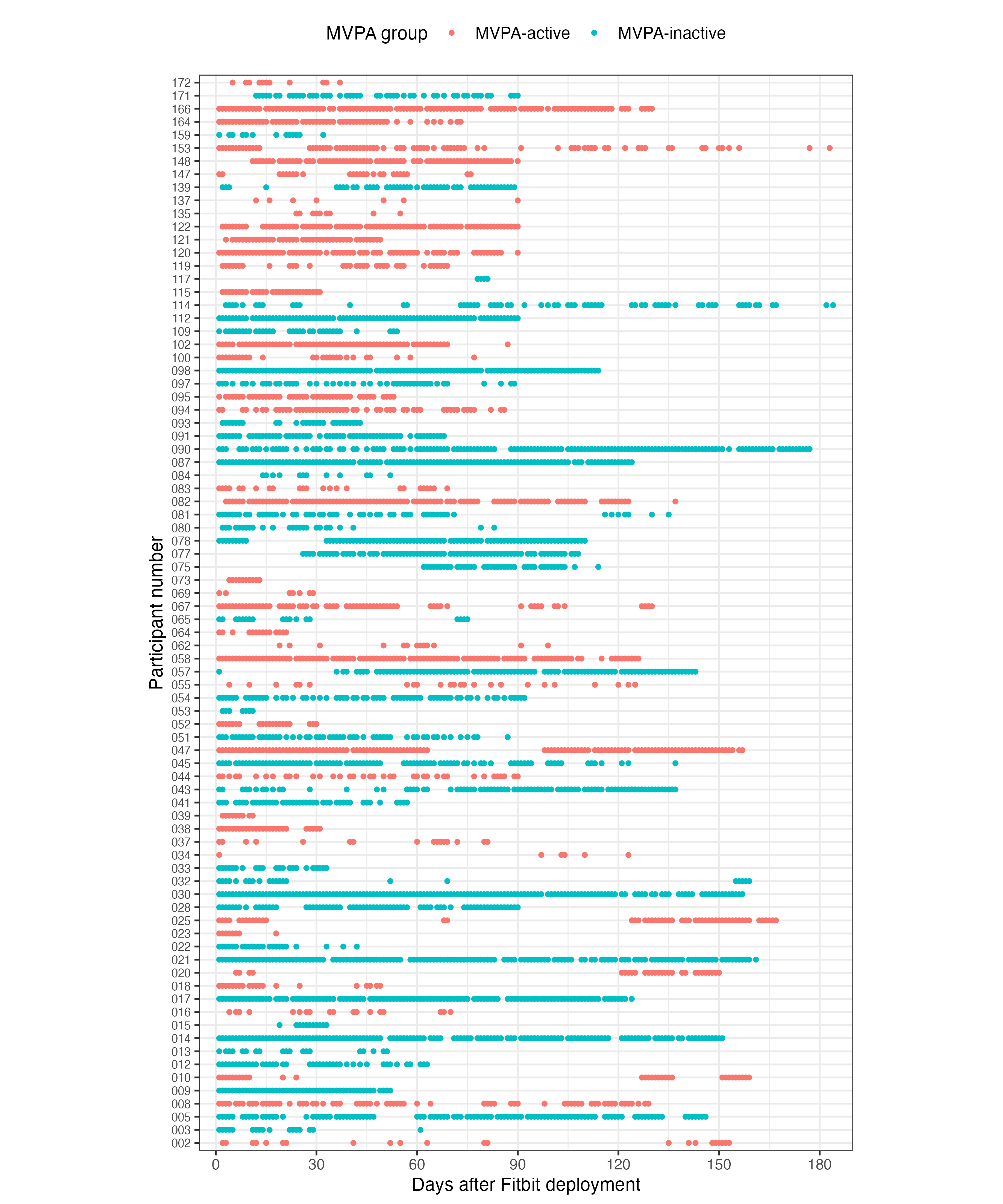

Supplement: Multimedia Appendix 3 [file formative_v9i1e77644_app3.png]
